# Genetic variants in *C1GALT1* are associated with gastric cancer risk by influencing immune infiltration

**DOI:** 10.7555/JBR.37.20230161

**Published:** 2024-05-29

**Authors:** Mengfan Guo, Jingyuan Liu, Yujuan Zhang, Jingjing Gu, Junyi Xin, Mulong Du, Haiyan Chu, Meilin Wang, Hanting Liu, Zhengdong Zhang

**Affiliations:** 1 Departments of Environmental Genomics and Genetic Toxicology, the Key Laboratory of Modern Toxicology of Ministry of Education, Center for Global Health, Jiangsu Key Laboratory of Cancer Biomarkers, Prevention and Treatment, Collaborative Innovation Center for Cancer Personalized Medicine, School of Public Health; Institute of Clinical Research, the Affiliated Taizhou People's Hospital of Nanjing Medical University, Nanjing Medical University, Nanjing, Jiangsu 211166, China; 2 Departments of Environmental Genomics and Genetic Toxicology, the Key Laboratory of Modern Toxicology of Ministry of Education, Center for Global Health, Jiangsu Key Laboratory of Cancer Biomarkers, Prevention and Treatment, Collaborative Innovation Center for Cancer Personalized Medicine, School of Public Health, Nanjing Medical University, Nanjing, Jiangsu 211166, China; 3 Department of Bioinformatics, School of Biomedical Engineering and Informatics, Nanjing Medical University, Nanjing, Jiangsu 211166, China; 4 Department of Biostatistics, Center for Global Health, School of Public Health, Nanjing Medical University, Nanjing, Jiangsu 211166, China

**Keywords:** O-glycosylation, genetic variants, immune status, gastric cancer

## Abstract

Core 1 synthase glycoprotein-N-acetylgalactosamine 3-β-galactosyltransferase 1 (C1GALT1) is known to play a critical role in the development of gastric cancer, but few studies have elucidated associations between genetic variants in *C1GALT1* and gastric cancer risk. By using the genome-wide association study data from the database of Genotype and Phenotype (dbGAP), we evaluated such associations with a multivariable logistic regression model and identified that the rs35999583 G>C in *C1GALT1* was associated with gastric cancer risk (odds ratio, 0.83; 95% confidence interval [CI], 0.75–0.92; *P* = 3.95 × 10 ^−4^). *C1GALT1* mRNA expression levels were significantly higher in gastric tumor tissues than in normal tissues, and gastric cancer patients with higher *C1GALT1* mRNA levels had worse overall survival rates (hazards ratio, 1.33; 95% CI, 1.05–1.68; *P*_log-rank_ = 1.90 × 10 ^−2^). Furthermore, we found that *C1GALT1* copy number differed in various immune cells and that *C1GALT1* mRNA expression levels were positively correlated with the infiltrating levels of CD4 ^+^ T cells and macrophages. These results suggest that genetic variants of *C1GALT1* may play an important role in gastric cancer risk and provide a new insight for *C1GALT1* into a promising predictor of gastric cancer susceptibility and immune status.

## Introduction

Gastric cancer is the fourth leading cause of morbidity and a major health burden in China ^[ 1] ^. In recent years, the incidence of gastric cancer has decreased because of changes in lifestyles, including improved food storage methods, increased intake of fruits and vegetables, and treatment of *Helicobacter pylori* infection ^[ 2– 3] ^. However, the overall survival (OS) for patients with gastric cancer is still poor ^[ 4] ^. Most gastric cancer patients were already in advanced stages at the time of diagnosis ^[ 5] ^, making gastric cancer a major health challenge. It is known that cancer progression and metastasis may be affected by genetic factors of the host, such as single nucleotide polymorphism (SNP), which is a common genetic variation ^[ 6]^ and is often used to evaluate both cancer susceptibility and prognosis ^[ 7] ^. Multiple SNPs associated with gastric cancer susceptibility have been found in genome-wide association studies (GWASs), providing important tools for further exploring the etiology of gastric cancer ^[ 8] ^.

Abnormal glycosylation is often associated with clinically significant pathogenesis ^[ 9– 11]^ and is a hallmark of cancers, leading to the formation of tumor-associated glycans or glycoproteins ^[ 12] ^. O-glycan is the major form of glycosylation modification and a major component of gastric mucus ^[ 13] ^. GalNAc-type O-glycosylation is the most common type of O-glycosylation ^[ 14] ^. Core 1 synthase glycoprotein-N-acetylgalactosamine 3-β-galactosyltransferase 1 (C1GALT1) plays a key role in GalNAc-type O-glycosylation and is associated with the progression and prognosis of various types of cancer ^[ 15] ^. It has been reported that *C1galt1* ^*−/−*^ mice are more susceptible to gastric cancer ^[ 16] ^, and C1GALT1 expression affects gastric cancer progression ^[ 17] ^. However, associations between genetic variants in *C1GALT1* and gastric cancer risk have not been reported. On the one hand, C1GALT1 expression influences inflammatory and immune-mediated diseases ^[ 18] ^; on the other hand, the immune microenvironment influences the treatment and prognosis of gastric cancer patients ^[ 19] ^. For example, one study has demonstrated an interaction between genetic variants in *C1GALT1* and IgA1 levels ^[ 20] ^, indicating that *C1GALT1* may be associated with immunity. Thus, both genetic variants in *C1GALT1* and immune status may play a role in the development of gastric cancer.

In the present study, we evaluated the associations between genetic variants in the O-glycosylation key gene *C1GALT1* and gastric cancer risk, and validated possible underlying immunoregulatory mechanisms.

## Subjects and methods

### Study subjects

We used the GWAS data from the database of Genotype and Phenotype (dbGAP, phs000361. v1. p1), and the detailed information on the recruitment and characteristics of the subjects was previously reported ^[ 21] ^. Cases and controls were frequency-matched on age and sex. This GWAS study obtained informed consent from the subjects, which was approved by their Institutional Review Boards, and the Special Institutional Review Board of the National Cancer Institute.

### SNP selection and genotyping

To investigate the association between *C1GALT1* and gastric cancer risk, we performed the Multi-marker Analysis of GenoMic Annotation (MAGMA) using GWAS data. Firstly, we extracted SNPs located in the *C1GALT1* region from the 1000 Genomes Project and retained SNPs that met the following inclusion criteria for quality control: minor allele frequency > 0.05, *P*-value for Hardy-Weinberg equilibrium > 0.05, and call rate > 95%. Then, we selected the independent SNPs after the pairwise linkage disequilibrium analysis ( *r* ^2^ < 0.8) for further analysis. Finally, we used HaploReg v4.1 and RegulomeDB to predict potential functions of SNPs, and we used SNPs with RegulomeDB scores ≤ 5.

### *In silico* functional annotation

We used the RegulomeDB ( http://RegulomeDB.org/), HaploReg v4.1 ( https://pubs.broadinstitute.org/mammals/haploreg/haploreg.php), and FAVOR ( http://favor.genohub.org) websites to predict potential functions of SNPs. We subsequently conducted an analysis using 3DSNP v2.0 ( https://omic.tech/3dsnpv2/) to predict the enhancer states of SNPs. Furthermore, we employed the Human TFDB ( http://bioinfo.life.hust.edu.cn/HumanTFDB/) and JASPAR 2022 database ( https://jaspar.genereg.net/) to predict transcription factors. Histone modification peaks were derived from the Cistrome Data Browser ( http://cistrome.org/db/#/) and visualized by the WashU Epigenome Browser ( http://epigenomegateway.wustl.edu/). The histone modifications of risk SNPs in multiple cells and high-throughput chromosome conformation capture (Hi-C) interacting maps were revealed through the University of California Santa Cruz (UCSC) Genome Browser ( http://genome.ucsc.edu/). To determine the associations between genetic variants and various traits, we used the MRC-IEU OpenGWAS database for the phenome-wide association study (PheWAS, https://gwas.mrcieu.ac.uk/) ^[ 22] ^.

### Gene expression

We first analyzed the mRNA expression levels of *C1GALT1* using datasets from The Cancer Genome Atlas (TCGA, https://cancergenome.nih.gov/) and the Gene Expression Omnibus (GEO, https://www.ncbi.nlm.nih.gov/gds/). We further examined the protein expression levels of C1GALT1 in gastric cancer tissues using the data from the Human Protein Atlas (HPA, https://www.proteinatlas.org/) ^[ 23] ^. We also assessed the effects of expression levels of the selected genes on the survival of gastric cancer patients through the Kaplan-Meier Plotter ( https://kmplot.com/analysis/).

### Immunohistochemical (IHC) staining

To further examine the protein expression levels of C1GALT1 in gastric cancer tissues, we performed IHC staining to detect C1GALT1 expression levels (1∶100; Proteintech, 27569-1-AP, Wuhan, China). The tumor samples were obtained from three patients with gastric cancer from the Affiliated Huai'an No. 1 People's Hospital of Nanjing Medical University. Briefly, the tissues were fixed with 4% paraformaldehyde at room temperature. After paraffin embedding, the tissue paraffin blocks were processed as 4 µm sections. The prepared slides were then deparaffinized by xylene and rehydrated with decreased concentrations of ethanol. After antigen retrieval by high-pressure heat treatment, slides were treated with 3% H _2_O _2_ for 10 min. Then, the tissue slides were blocked in 10% goat serum for 30 min and incubated with primary antibodies overnight at 4 ℃. On the next day, the slides were washed with TBST three times. HRP-conjugated secondary antibody was used and incubated at 37 ℃ for 1 h. Then, the slides were washed with TBST three times, and were color-developed with 3,3′-diaminobenzidine (DAB). The slides were re-stained using hematoxylin for 30 s and then, the slides were differentiated for 10 s by using an acid alcohol differentiation solution. The slides were stained for 1 min with the rebluing solution, and then dehydrated and sealed with neutral resin. The images were analyzed by Pannoramic SCAN (3DHISTECH, Budapest, Hungary).

### The tumor microenvironment (TME) immunoregulation analysis

We used the Tumor Immune Estimation Resource (TIMER, http://timer.cistrome.org/) website for immunoregulation analysis ^[ 24]^ to assess the correlations between the expression levels of the target gene and the abundance of TME-infiltrating immune cells. We further estimated the Spearman correlation coefficients between immune markers in immune cell types and target genes, along with their statistical significance. Tumor purity was adjusted for what has been proven to influence the analysis of immune infiltration.

### Statistical analysis

To evaluate the associations between genetic variants and gastric cancer risk with odds ratios (ORs) and their 95% confidence intervals (CIs), we used the multivariable logistic regression model with adjustment of age and sex. We also performed the false discovery rate correction to correct for multiple comparisons. We further assessed the *C1GALT1* expression levels (log _2_-transformed) between gastric tumor tissues and adjacent normal tissues. This analysis was performed using the data from both TCGA and GEO databases, employing Student's *t*-test or a two-tailed Mann-Whitney *U* test for statistical analysis. Pearson's analysis was used to analyze the correlations between the C1GALT1 expression and mRNA levels of transcription factors. All statistical analyses were performed by R 4.0.5 and PLINK 1.90, with a *P*-value < 0.05 considered statistically significant.

## Results

### Correlation of SNPs with susceptibility to gastric cancer

**Figure 1:**
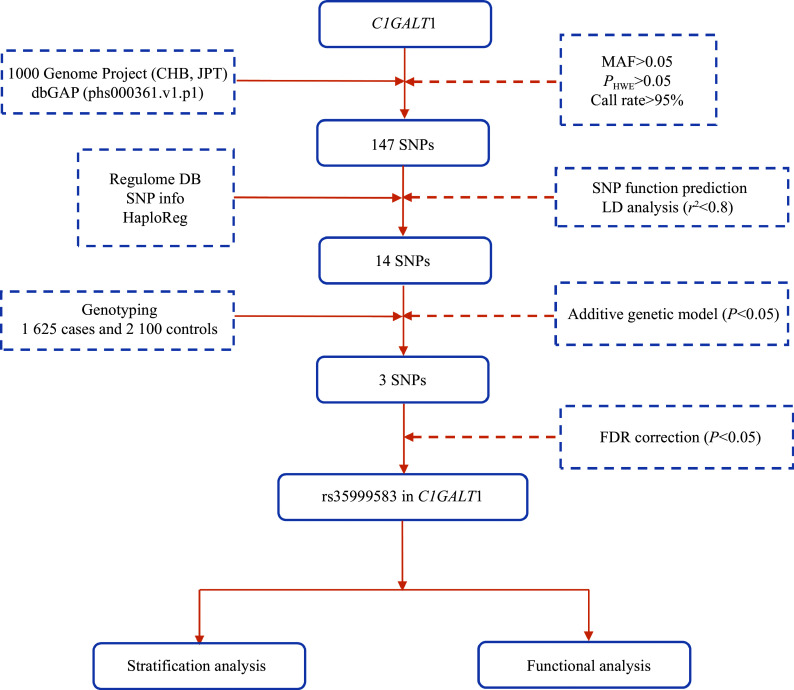
A flow diagram for selecting SNPs in *C1GALT1*.

The flowchart of SNP selection is shown in ***Fig. 1***. Through the MAGMA tool based on GWAS data, *C1GALT1* expression levels were associated with gastric cancer risk ( *P* = 2.95 × 10 ^−2^, ***Supplementary Table 1***, available online). We extracted SNPs within *C1GALT1*, and 147 SNPs remained after quality control. After the linkage disequilibrium and *in silico* analysis, a total of 14 independent SNPs ( *r* ^2^ < 0.8) were selected for genotyping, and functional annotations of the tagged SNPs are shown in ***Supplementary Table 2*** (available online). Three SNPs were significantly associated with gastric cancer risk, but only rs35999583 remained significant after false discovery rate correction ( *P* = 5.52 × 10 ^−3^, ***Table 1***).

**Table 1:** Associations of three significant SNPs in *C1GALT1* with gastric cancer risk

Chr	SNP	Position	Gene	Allele ^a^	MAF-controls	MAF-cases	OR (95% CI) ^b^	*P* ^b^	*P* ^c^
7	rs35999583	7247368	*C1GALT1*	G/C	0.33	0.29	0.83 (0.75–0.92)	3.95×10 ^−4^	5.52×10 ^−3^
7	rs73049974	7282894	*C1GALT1*	G/A	0.21	0.19	0.87 (0.77–0.97)	1.48×10 ^−2^	1.03×10 ^−1^
7	rs11764290	7284591	*C1GALT1*	C/T	0.28	0.31	1.11 (1.01–1.23)	3.93×10 ^−2^	1.84×10 ^−1^
^a^Reference allele/effect allele. ^b^Adjusted for age and sex in the additive model. ^c^ *P* after false discovery rate correction.Abbreviations: Chr, chromosome; SNP, single nucleotide polymorphism; MAF, minor allele frequency; OR, odds ratio; CI, confidence interval.									

To further analyze the association between the rs35999583 and gastric cancer risk, three genetic models were used ( ***Table 2***). Specifically, compared with the GG genotype, the rs35999583 GC and CC genotypes were associated with 15% (OR = 0.85; 95% CI: 0.74–0.98, *P* =2.50 × 10 ^−2^) and 34% decreased gastric cancer risk (OR = 0.66; 95% CI: 0.52–0.85, *P* =1.00 × 10 ^−3^), respectively. In the dominant model, individuals with the GC/CC genotypes had a decreased gastric cancer risk (OR = 0.82; 95% CI: 0.71–0.93, *P* =2.77 × 10 ^−3^), while in the recessive model, subjects with the CC genotype had a 28% decreased gastric cancer risk, compared with the GC/GG genotypes (OR = 0.72; 95% CI: 0.57–0.91, *P* =5.34 × 10 ^−3^).

**Table 2:** Association between rs35999583 and gastric cancer risk

SNP	Cases			Controls		OR (95% CI)	*P*	OR (95% CI) ^a^	*P* ^a^
	*n*	%		*n*	%				
rs35999583									
GG	789	50.4		911	45.4	1.00		1.00	
GC	654	41.8		884	44.0	0.85 (0.74–0.98)	2.62×10 ^–2^	0.85 (0.74–0.98)	2.50×10 ^−2^
CC	122	7.8		212	10.6	0.66 (0.52–0.85)	9.43×10 ^–4^	0.66 (0.52–0.85)	1.00×10 ^–3^
Additive model						0.83 (0.75–0.92)	3.90×10 ^–4^	0.83 (0.75–0.92)	3.95×10 ^–4^
Dominant model						0.82 (0.72–0.93)	2.87×10 ^–3^	0.82 (0.71–0.93)	2.77×10 ^–3^
Recessive model						0.72 (0.57–0.90)	4.97×10 ^–3^	0.72 (0.57–0.91)	5.34×10 ^–3^
^a^Adjusted for age and sex in the logistic regression model.Abbreviations: OR, odds ratio; CI, confidence interval.									

### Stratified analysis of rs35999583 with gastric cancer risk

We performed stratified analyses in the additive genetic model with adjustment of age and sex ( ***Supplementary Table 3***, available online). The variant genotypes were associated with the risk of gastric cancer in the subgroups of age (OR = 0.82, 95% CI: 0.71–0.94, *P* = 5.06 × 10 ^–3^ for age > 60; OR = 0.85, 95% CI: 0.73–0.99, *P* = 3.12 × 10 ^−2^ for age ≤ 60) and sex (OR = 0.87, 95% CI: 0.78–0.98, *P* = 2.62 × 10 ^−2^ for males; OR = 0.71, 95% CI: 0.58–0.88, *P* =1.25 × 10 ^−3^ for females). There were no statistically significant differences in the distributions of allele frequencies in the subgroups of age and sex.

### Potential regulatory function of rs35999583

We further performed the *in* *silico* analysis to predict potential functions of the candidate SNP. Based on the prediction, rs35999583 was located in the DNAse sensitive and motif changed as well as histone modification region ( ***Supplementary Tables 4*** and ***5***, available online). For gastric tumor cells, we conducted functional annotations by using publicly available epigenomic data from the Cistrome Data Browser, which were visualized by the WashU genome browser. As shown in ***Supplementary Fig. 1*** (available online), the region of rs35999583 was enriched with histone modification peaks including histone H3 lysine 4 monomethylation (H3K4me1) and histone H3 lysine 27 acetylation (H3k27ac), which was similar to those identified in the UCSC Genome Browser ( ***Supplementary Fig. 2 ***, available online).

**Figure 2:**
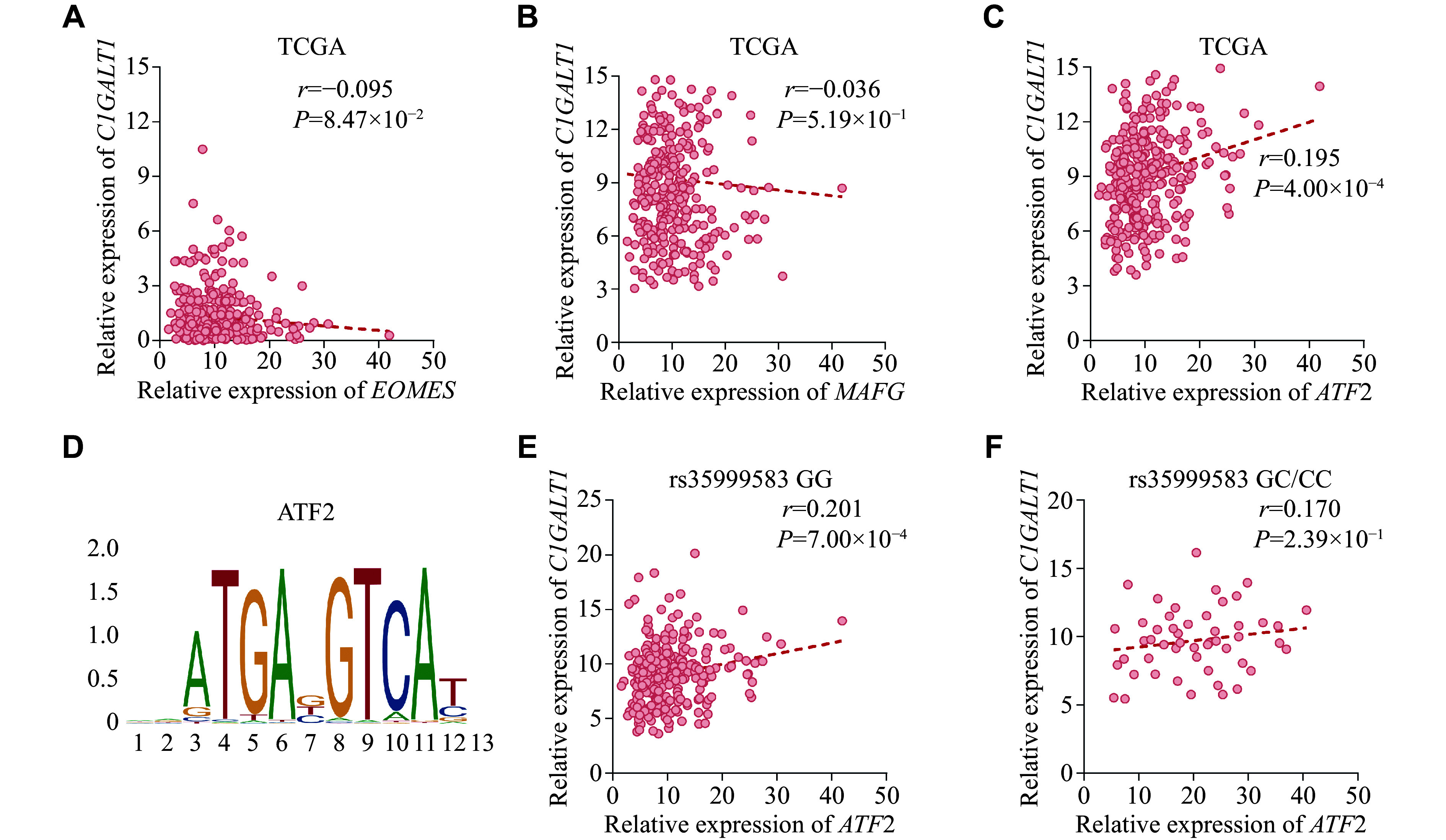
The potential regulatory role of SNP rs35999583 on *C1GALT1*.

We also performed a motif analysis using the Human TFDB and scanned these transcription factor motifs in the JASPAR database ( ***Supplementary Tables 6*** and ***7***, available online), and EOMES, MAFG, and ATF2 were predicted to bind to rs35999583. We further evaluated the correlations between these candidate transcription factors and C1GALT1, and found that the expression levels of *ATF2* were significantly correlated with that of *C1GALT1* ( ***Fig. 2A***– ***2D***). This may explain the risk effect of the target SNP. Moreover, based on the TCGA database, *ATF2* expression was positively correlated with *C1GALT1* expression ( *r* = 0.195, *P* = 4.00 × 10 ^−4^), and the correlation between *ATF2* expression and *C1GALT1* expression was weaker in patients carrying the risk allele than in those carrying the protective allele (for G allele: *r* = 0.201, *P* = 7.00 × 10 ^−4^; for C allele: *r* = 0.170, *P* = 2.39 × 10 ^−1^, ***Fig. 2E*** and ***2F***), suggesting that *C1GALT1* expression may be influenced by the binding to ATF2. In the subsequent PheWAS analysis for the target SNP, we found that the rs35999583 was associated with multiple diseases as shown in ***Supplementary Table 8*** (available online).

### *C1GALT1* expression and survival analysis

**Figure 3:**
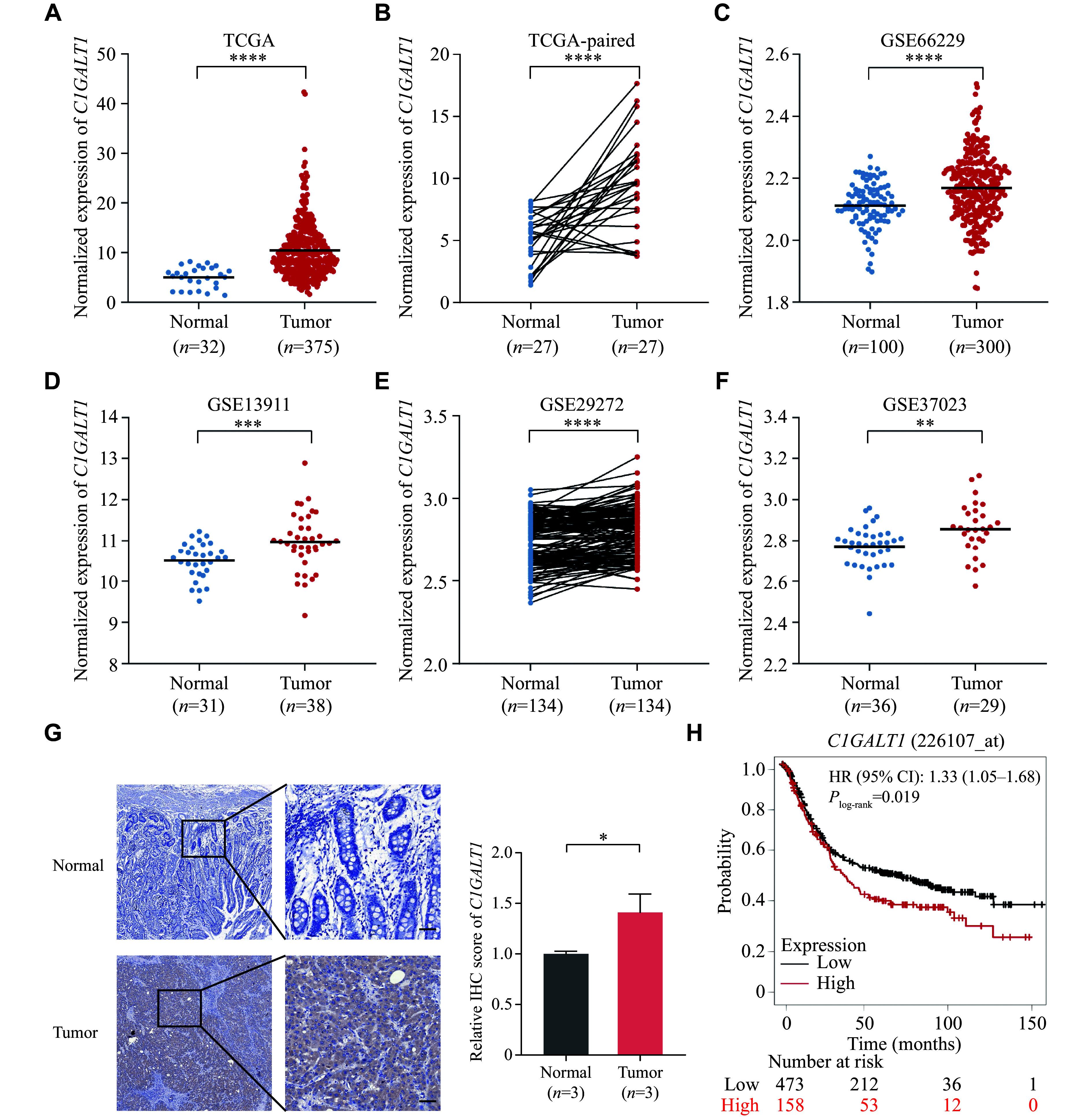
*C1GALT1* had significantly higher expression levels in gastric tumor tissues than in adjacent normal tissues.

We then used the TCGA and GEO databases to assess the *C1GALT1* expression in gastric tumor tissues and adjacent normal tissues. We found that the expression levels of *C1GALT1* were significantly higher in gastric tumor tissues than in adjacent normal tissues ( ***Fig. 3A*** and ***3B***) in the data obtained from the TCGA database, and these results were further validated by the analyses of the paired tissues in the GEO database (GSE66229, GSE13911, GSE29272, and GSE37023, respectively) ( ***Fig. 3C***– ***3F***). We also found that *C1GALT1* was moderately expressed in gastric tissues ( ***Supplementary Fig. 3 ***, available online). In addition, the protein levels of C1GALT1 showed a higher expression in gastric tumor tissues than in adjacent normal tissues ( ***Fig. 3G***).

We also evaluated the effect of *C1GALT1* on gastric cancer survival by using the online bioinformatics tool Kaplan-Meier Plotter, and found that patients with gastric cancer, who carried high *C1GALT1* mRNA levels, had a prominent poorer OS (hazards ratio = 1.33, 95% CI: 1.05–1.68), *P*_log-rank_ = 1.90 × 10 ^−2^, ***Fig. 3H***) than those with low *C1GALT1* mRNA levels.

### The potential role of *C1GALT1* as an immunoregulatory gene in the TME

**Figure 4:**
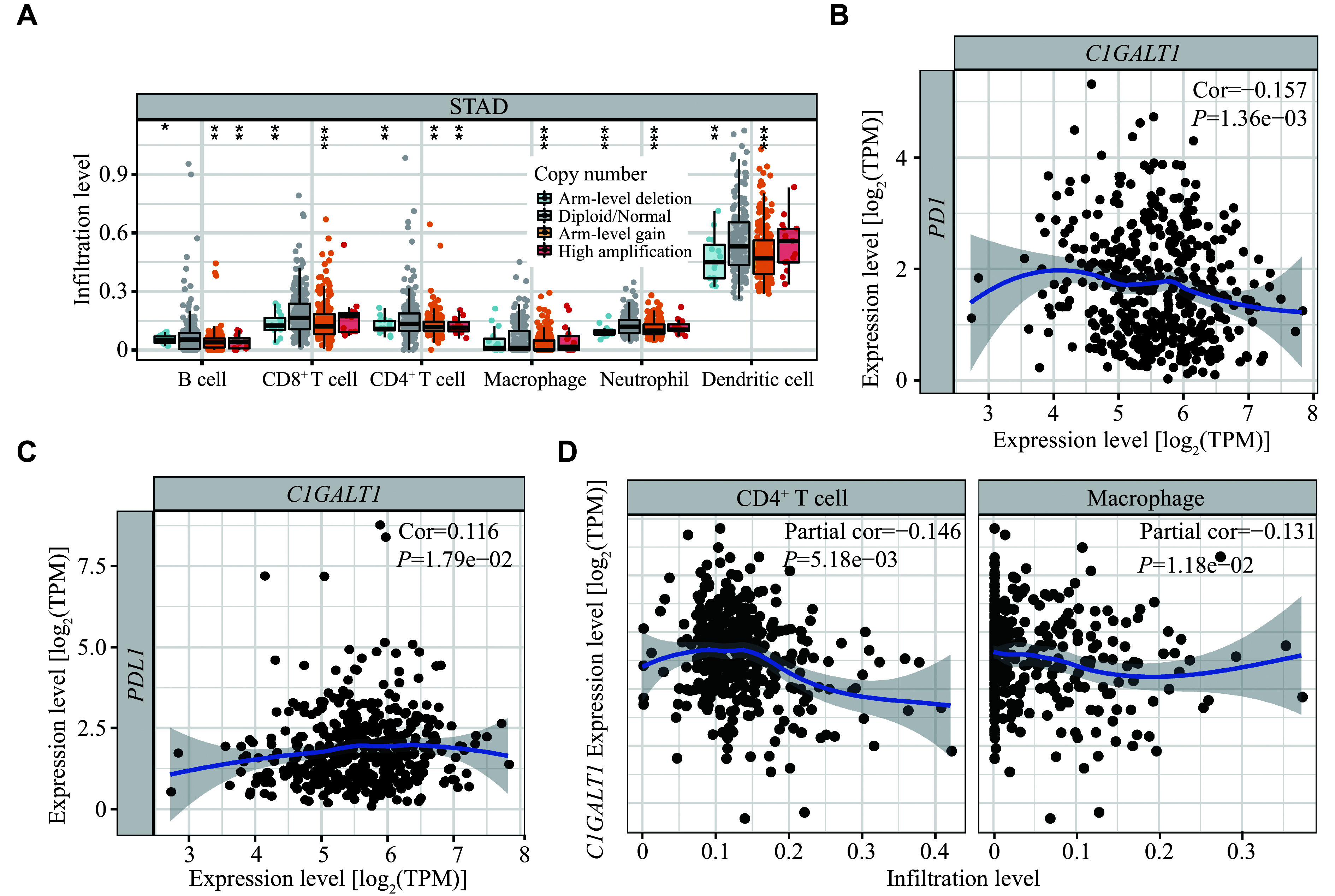
The immune infiltration analyses of *C1GALT1* in STAD.

C1GALT1 is known to affect immune status, and mutations in *C1GALT1* occur across multiple cancer types ( ***Supplementary Fig. 4 ***, available online). Therefore, we assessed the correlations between *C1GALT1* expression and infiltration levels of immune cells to validate potential functions of *C1GALT1* in tumor immune regulation using the TIMER database. In gastric adenocarcinoma data, the harm-level deletion or gain of *C1GALT1* was associated with the infiltration levels of various immune cells ( ***Fig. 4A***). We further assessed correlations between *C1GALT1* expression and hub T cell checkpoint status, and found that *C1GALT1* expression was significantly correlated with programmed cell death protein 1 ( *PD1*) and programmed cell death-ligand 1 ( *PDL1*) ( ***Fig. 4B*** and ***4C***). The correlations between *C1GALT1* expression and immune signatures were also evaluated, and immune cells were identified by gene markers. In gastric adenocarcinoma data, 19 of the 42 T cell markers were associated with *C1GALT1* expression ( ***Supplementary Table 9***, available online), and *C1GALT1* expression was positively correlated with the levels of infiltrating of CD4 ^+^ T cells and macrophages ( ***Fig. 4D***). Taken together, these findings suggest that *C1GALT1* expression was associated with immune infiltration status in gastric tumor tissues.

## Discussion

Abnormal glycosylation is involved in carcinogenesis and tumor progression ^[ 25] ^, and genetic variants in *C1GALT1* are known to interact with immunity ^[ 20] ^. In the present study, we conducted a case-control study to investigate the associations between genetic variants in *C1GALT1* and risk of gastric cancer, and also assessed the association between *C1GALT1* mRNA levels and immune status. We found that the rs35999583 G>C change was associated with a significantly reduced gastric cancer risk. Moreover, the copy number variations of *C1GALT1* were associated with the infiltration degree of immune cells. We also observed the associations of *C1GALT1* expression levels with immune marker genes or OS time. These results suggest that *C1GALT1* may be a promising predictor of gastric cancer susceptibility and play a key role in immune regulation.

The crucial step of GalNAc-type O-glycosylation is affected by C1GALT1, a main contributor to oncogenesis ^[ 26] ^. It was reported that the *C1galt1* ^*−/−*^ mice developed diseases including gastritis and gastric cancer, revealing a critical role of O-glycosylation in gastric homeostasis ^[ 16] ^. The gastric carcinogenesis is a multi-step process, including chronic non-atrophic gastritis ^[ 27]^ and gastric tissues interface with a hostile luminal environment, such as chemical agents, microorganisms, and mechanical stress, through gastric gland-derived oligosaccharide-rich mucins ^[ 28– 29] ^. The development of gastric cancer is also influenced by genetic factors ^[ 30] ^. However, few studies have reported the associations between genetic variants in *C1GALT1* and gastric cancer risk.

In the present study, we found that *C1GALT1* was associated with gastric cancer risk through the gene-based analysis, and that rs35999583 within *C1GALT1* was significantly associated with gastric cancer risk. Furthermore, carriers of the rs35999583 C allele had a reduced gastric cancer risk, independent of age and sex. Thus, our results indicate that *C1GALT1* rs35999583 may be a risk factor for gastric cancer.

SNP rs35999583 is located in the intron region enriched with H3K4me1 and H3K27ac, both of which are active enhancers, suggesting a possible effect of rs35999583 on *C1GALT1* expression by influencing transcription factor binding. Our results suggest that the transcription factor ATF2 may bind to rs35999583 and that genotypic alterations of rs35999583 may affect the correlations between ATF2 and *C1GALT1* expression levels, predicting that the altered genotype may affect downstream transcription. Subsequent PheWAS analysis showed that *C1GALT1* rs35999583 was associated with multiple disease phenotypes, indicating that rs35999583 plays an important role in disease development and progression.

Aberrant glycosylation has an effect on tumor progression and chemoresistance in a variety of cancers, and C1GALT1 is the key enzyme to control GalNAc-type O-glycosylation ^[ 31] ^. O-glycosylation mediated by C1GALT1 affects the invasion and metastasis of a variety of tumors *via* regulating receptor tyrosine kinase, and the expression of C1GALT1 is associated with a poor prognosis of cancer patients ^[ 26, 32] ^, suggesting that C1GALT1 plays a key role in the development and progression of cancer. Furthermore, we found that the *C1GALT1* expression levels were higher in gastric tumor tissues than in adjacent normal tissues in our clinical samples but not in the public data ( ***Supplementary Fig. 5***, available online), which is probably because of the individual differences. In addition, patients with higher expression levels of *C1GALT1* had a worse OS time. These results suggest that C1GALT1 may play an important role in gastric cancer development and progression.

The activity of C1GALT1 was also reported to be associated with immune-mediated diseases in humans ^[ 33] ^. In the present study, we found that the degree of infiltration of various immune cells was associated with *C1GALT1* copy number variations, suggesting that genetic variants also influence the immune status. It is well known that cells in the TME communicate *via* the membrane-bound and secreted proteins, which are mostly glycosylated and thus affected by C1GALT1-mediated glycosylation. We found that the expression of *C1GALT1* was positively correlated with the levels of infiltrating of CD4 ^+^ T cells and macrophages in gastric cancer, indicating that C1GALT1 may govern crosstalk with macrophages and cytotoxic T lymphocytes, which was consistent with those findings in a previous study ^[ 34] ^. Immune checkpoint blockade is now established as a treatment for gastric cancer, and the treatment targeting PD-1 and PD-L1 significantly improves the survival of gastric cancer patients ^[ 5] ^. In the present study, we found that *C1GALT1* expression was significantly correlated with the expression levels of *PD1* and *PDL1*, suggesting that C1GALT1 expression may be associated with immune infiltration and thus may play an important role in immune escape in the microenvironment of gastric cancer.

Some potential limitations exist in the present study. Data on smoking, drinking, follow-up time, and chemotherapy were not collected in the study, and the observed associations between SNPs in *C1GALT1* and gastric cancer susceptibility need to be further verified in an independent population. Although the variant genotypes of *C1GALT1* were associated with the risk of gastric cancer, the exact underlying molecular mechanism is still unclear, which requires future experimental studies and molecular biology investigations.

Overall, we demonstrated significant associations between genetic variants in *C1GALT1* and gastric cancer risk, and also found that *C1GALT1* expression was associated with immune infiltration status, indicating that *C1GALT1* may be a potential independent biomarker for gastric cancer risk and immune regulation.

## SUPPLEMENTARY DATA

Supplementary data to this article can be found online.
