# Supplementary material for: Genetic variants in C1GALT1 are associated with gastric cancer risk by influencing immune infiltration
Source: J Biomed Res. 2024 May 29;38(4):348–57. doi: 10.7555/JBR.37.20230161 (PMC11300523; doi:10.7555/JBR.37.20230161)
Supplement: Supplementary file 1 — Supplementary data to this article can be found online. [file jbr-38-4-348-S1.pdf]

Available online at www.jbr-pub.org.cn

Open Access at PubMed Central

Journal of Biomedical Research, 2024 0(000): 1–7

JBR

Original Article

Genetic variants in *C1GALT1* are associated with gastric cancer risk and by influencing immune infiltration

Mengfan Guo[1,2,△], Jingyuan Liu[1,2,△], Yujuan Zhang[1,2,△], Jingjing Gu[1], Junyi Xin[1,3], Mulong Du[1], Haiyan Chu[1], Meilin Wang[1], Hanting Liu[1,✉], Zhengdong Zhang[1,2,✉]

[1]*Departments of Genetic Toxicology and Environmental Genomics, the Key Laboratory of Modern Toxicology of Ministry of Education, Center for Global Health, Jiangsu Key Laboratory of Cancer Biomarkers, Prevention and Treatment, Collaborative Innovation Center for Cancer Personalized Medicine, School of Public Health, Nanjing Medical University, Nanjing, Jiangsu 211166, China;*
[2]*The Affiliated Taizhou People's Hospital of Nanjing Medical University, Taizhou, Jiangsu 211103, China;*
[3]*Department of Bioinformatics, School of Biomedical Engineering and Informatics, Nanjing Medical University, Nanjing, Jiangsu 211166, China.*

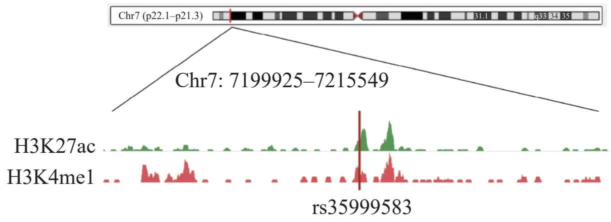

Supplementary Fig. 1 **Potential regulatory function of *C1GALT1* rs35999583.** Annotation for the region surrounding rs35999583 in gastric tumor cell (NCC-59), including histone H3 lysine 27 acetylation (H3K27ac) and histone H3 lysine 4 monomethylation (H3K4me1) modification peaks. The red line indicates the position of rs35999583.

△These authors contributed equally to this work.

✉Corresponding authors: Zhengdong Zhang and Hanting Liu, Department of Genetic Toxicology and Environmental Genomics, School of Public Health, Nanjing Medical University, 101 Longmian Avenue, Jiangning District, Nanjing, Jiangsu 211166, China. E-mails: drzdzhang@njmu.edu.cn (Zhang) and hantingliu@njmu.edu.cn (Liu).

Received: 12 July 2023; Revised: 09 December 2023; Accepted: 08 January 2024; Published online: 23 January 2024

CLC number: R735.2, Document code: A

The authors reported no conflict of interests.

This is an open access article under the Creative Commons Attribution (CC BY 4.0) license, which permits others to distribute, remix, adapt and build upon this work, for commercial use, provided the original work is properly cited.

© 2024 by Journal of Biomedical Research.

https://doi.org/10.7555/JBR.37.20230161

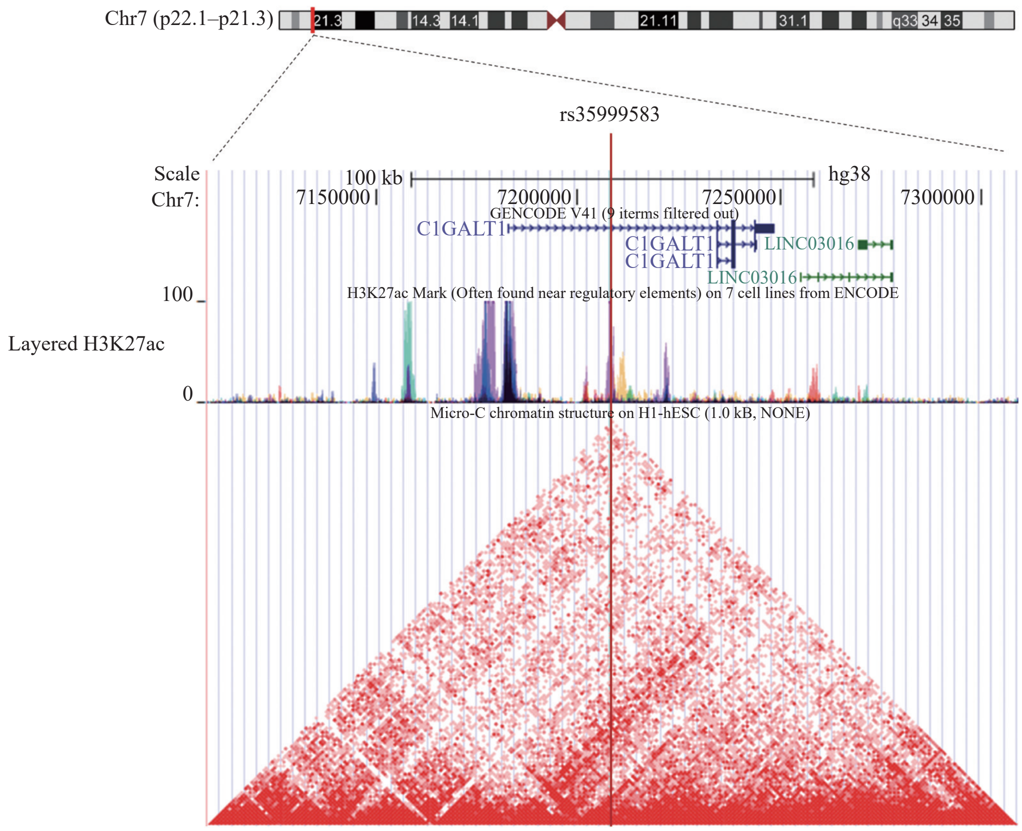

Supplementary Fig. 2 **The possible mechanisms of rs35999583.** The visualization of the enrichment of histone H3 lysine 27 acetylation (H3K27ac) with data from the University of California Santa Cruz Genome Browser (http://genome.ucsc.edu/) (upper) and high-throughput chromosome conformation capture (Hi-C) interacting maps for rs35999583 (lower).

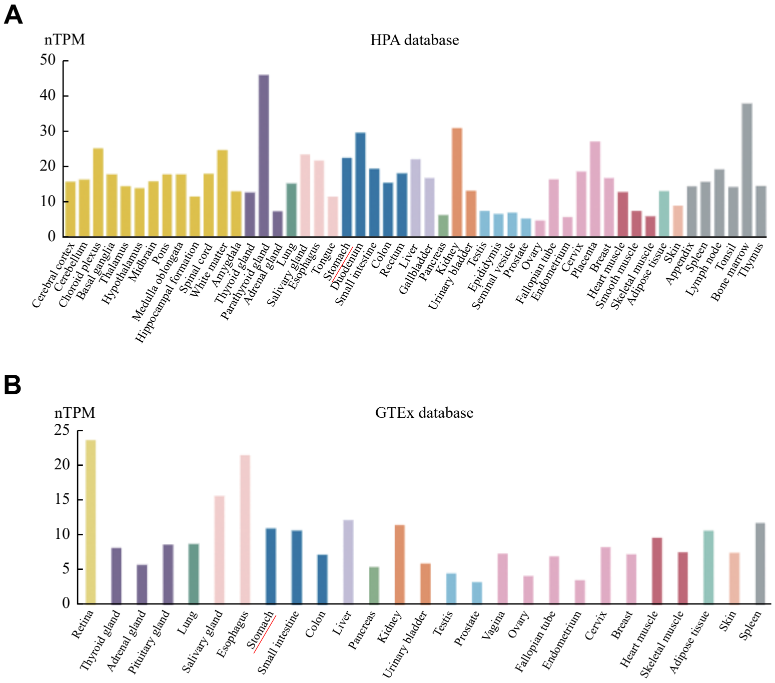

Supplementary Fig. 3 **The mRNA levels of *C1GALT1* in tissues of different organs.** A: The expression levels of *C1GALT1* in tissues of different organs using the Human Protein Atlas (HPA) website. B: The expression levels of *C1GALT1* in tissues of different organs using the Genotype-Tissue Expression (GTEx) project.

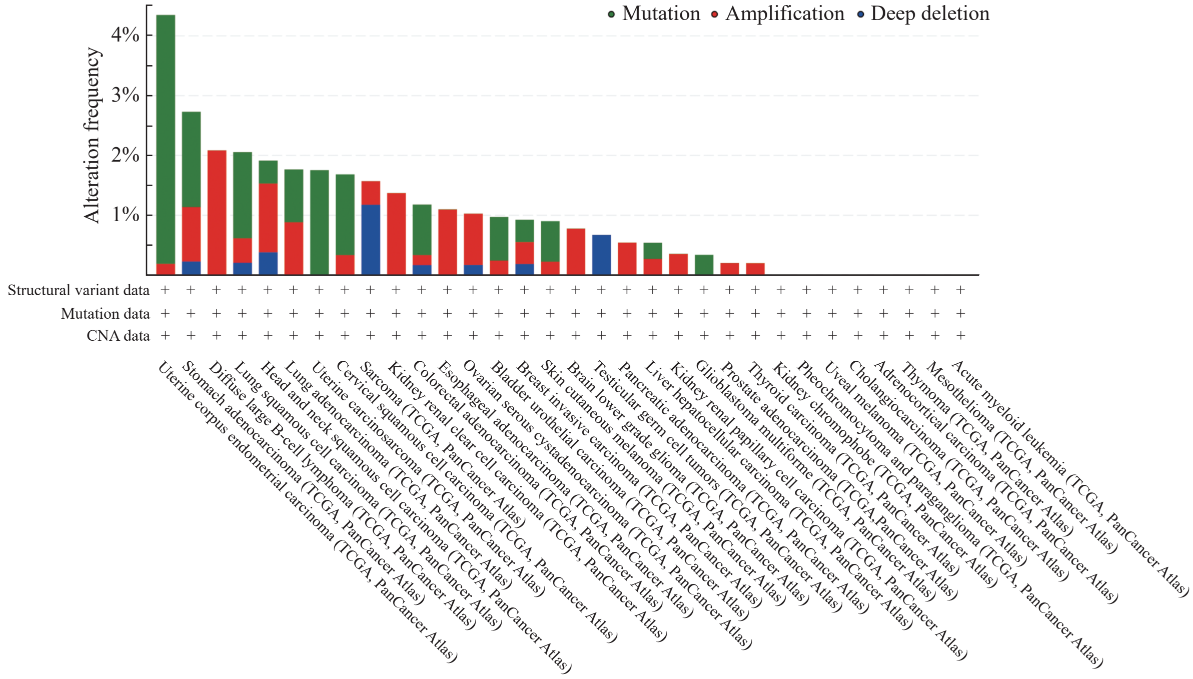

Supplementary Fig. 4 **The mutations in *C1GALT1* from multiple tumors.** Abbreviations: CNA, copy number alteration; TCGA, The Cancer Genome Atlas.

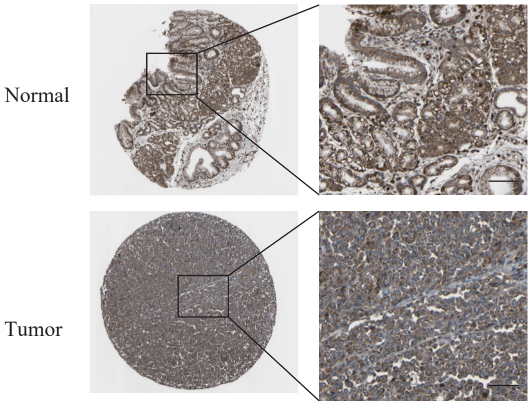

Supplementary Fig. 5 **Representative immunohistochemistry results of C1GALT using Human Protein Atlas dataset.** Scale bar, 50 μm.

Supplementary Table 1 **Gene-based analysis results by MAGMA**

Gene	Variants[a]	*Z*-stat[b]	*P*
C1GALT1	147	1.89	2.95×10^{-2}

[a]Number of variants annotated to the gene.
[b]Z-value for each gene.
Abbreviation: MAGMA, Multi-marker Analysis of GenoMic Annotation.

Supplementary Table 2 **Analysis for function annotation of the tagged SNPs**

SNPs	Chr	Position[a]	Allele[b]	RegulomeDB	Haploreg
rs35999583	7	7247368	G/C	5	Promoter histone marks, enhancer histone marks, DNAse, motifs changed
rs73049974	7	7282894	G/A	5	Enhancer histone marks, DNAse
rs11764290	7	7284591	T/C	4	Enhancer histone marks, DNAse, motifs changed
rs10253466	7	7250079	G/A	5	DNAse
rs4724963	7	7272034	C/T	4	Enhancer histone marks, motifs changed
rs10282358	7	7254866	C/G	5	–
rs138728158	7	7232606	G/A	1a	Enhancer histone marks, motifs changed
rs7780273	7	7250449	A/C	5	Promoter histone marks, enhancer histone marks, DNAse, motifs changed
rs2108788	7	7258901	T/C	3a	Enhancer histone marks, DNAse, motifs changed
rs11766771	7	7285180	G/A	5	Enhancer histone marks, DNAse, motifs changed

Supplementary Table 2 **Analysis for function annotation of the tagged SNPs** (Continued)

SNPs	Chr	Position[a]	Allele[b]	RegulomeDB	Haploreg
rs141913347	7	7268146	A/G	4	Promoter histone marks, enhancer histone marks, DNAse, motifs changed
rs11763876	7	7249747	A/G	5	Promoter histone marks, enhancer histone marks, DNAse, motifs changed
rs12164111	7	7230941	A/G	5	Enhancer histone marks, motifs changed
rs1008898	7	7273559	G/T	5	Motifs changed

[a]Based on NCBI build 37 of the human genome.
[b]Reference allele/affect allele.
–, no significant functions were reported for SNPs.

Supplementary Table 3 **Stratification analyses for rs35999583 genotypes and gastric cancer risk**

Variables	Genotypes (cases/controls)						OR (95% CI)[a]	*P*[a]	*P*[b]
	GG		GC		CC				
	n	%	*n*	%	*n*	%			
Age (years)									
>60	436/478	51.0 /45.6	352/459	41.2/43.8	67/111	7.80/10.6	0.82 (0.71−0.94)	5.06×10^{-3}	7.46×10^{-1}
≤60	353/433	49.7/45.2	302/425	42.6/44.3	55/101	7.7/10.5	0.85 (0.73−0.99)	3.12×10^{-2}	
Sex									
Male	604/627	49.7 /46.0	511/598	42.1/43.8	100/139	8.2/10.2	0.87 (0.78−0.98)	2.62×10^{-2}	9.28×10^{-1}
Female	185/284	52.9/44.1	143/286	40.9/44.5	22/73	6.2/11.4	0.71 (0.58−0.88)	1.25×10^{-3}	

[a]Adjusted for additive model adjusted for age and sex in the logistic regression model.
[b]Two-sided χ^2 test for the distributions of genotype and allele frequencies.
Abbreviations: OR, odds ratio; CI, confidence interval.

Supplementary Table 4 **The effect of rs35999583 *in silico* prediction**

SNP	RegulomeDB		3DSNP v2.0		HaploReg v4.1		
	Motifs	DNase	Enhancer	Promotor	Enhancer histone marks	Motifs	DNase
rs35999583	2	1 tissue	25 cell types	1	10 tissues	9	1 tissue

Supplementary Table 5 **The lists of functional scores specific to rs35999583 using FAVOR**

Function elements	Score
aPC-Conservation	10.523 994 49
aPC-Epigenetics-Active	17.609 320 05
aPC-Epigenetics-Repressed	2.140 621 923
aPC-Epigenetics-Transcription	10.543 045 62
aPC-Local-Nucleotide-Diversity	1.507 110 341
aPC-Mappability	4.774 092 068
aPC-Mutation-Density	0.682 996 71
aPC-Protein-Functions	2.969 487 03
aPC-Transcription-Factor	5.787 098 193
H3K4me1	20.72
H3K4me2	31.81
H3K4me3	9.58
H3K9ac	13.29
H3K9me3	10.72
H3K27ac	70.03
H3K27me3	4.3
H3K36me3	10.04
H3k79me2	64.66
H4k20me1	19.23

Supplementary Table 6 **The motif analysis of rs35999583 using the Human TFDB database**

Allele	TF	Score	Start	Stop	Matched sequence (5′-3′)
G	RELA	13.15	47	68	TGTGGTAATTCTGGAAATTAGG
	AIRE	11.64	41	66	ATTTAATGTGGTAATTCTGGAAATTA
	ATF2	11.14	45	56	AATGTGGTAATT
C	SMAD1	11.95	42	55	TTTAATGTGCTAAT
	MEF2A	11.48	47	62	TGTGCTAATTCTGGAA
	RELA	11.25	47	68	TGTGCTAATTCTGGAAATTAGG
	MAFG	8.66	44	64	TAATGTGCTAATTCTGGAAAT
	FOSL1	−2.89	47	62	TGTGCTAATTCTGGAA
	EOMES	10.83	42	57	TTTAATGTGCTAATTC
	STAT1	3.11	49	69	TGCTAATTCTGGAAATTAGGT

Supplementary Table 7 **The motif analysis of rs35999583 using the JASPAR database**

Allele	TF	Score	Start	End	Predicted sequence (5′-3′)
G	EOMES	7.676 674	45	57	AATGTGCTAATTC
C	MAFG	11.504 09	45	65	AATGTGCTAATTCTGGAAATT
	ATF2	8.559 89	44	56	TAATGTGGTAATT

Supplementary Table 8 **The PheWAS analysis for rs35999583**

ID	Trait	*P*	*n*
ukb-b-20544	Nervous feelings	2.40e−04	450 700
ukb-b-11348	Bread intake	3.40e−04	452 236
ukb-e-104110_AFR	Broad bean intake	4.08e−04	NA
ukb-b-4043	Mushroom intake	4.90e−04	64 949
ukb-a-50	Nervous feelings	5.23e−04	328 725
ebi-a-GCST006948	Feeling nervous	6.58e−04	373 121
ukb-b-2122	Ankle spacing width (left)	8.50e−04	146 226

Abbreviations: PheWAS, phenome-wide association study; NA, not available.

Supplementary Table 9 **Correlation analysis between *C1GALT1* and marker genes in immune cells using TIMER**

Description	Gene markers	STAD			
		Unadjusted by purity		Adjusted by purity	
		Cor	*P*	Cor	*P*
CD8+ T cell	CD8A	−0.107	$\mathbf{2.89{\times}10^{-2}}$	−0.102	$\mathbf{4.64{\times}10^{-2}}$
	CD8B	−0.114	$\mathbf{2.01{\times}10^{-2}}$	−0.107	$\mathbf{3.65{\times}10^{-2}}$
T cell (general)	*CD3D*	−0.097	$\mathbf{4.88{\times}10^{-2}}$	−0.084	$\mathbf{1.01{\times}10^{-2}}$
	CD3E	−0.089	$7.01{\times}10^{-2}$	−0.082	$\mathbf{1.12{\times}10^{-2}}$
	CD2	−0.025	$6.07{\times}10^{-1}$	−0.007	$8.91{\times}10^{-1}$
B cell	*CD19*	−0.148	$\mathbf{2.56{\times}10^{-3}}$	−0.134	$\mathbf{8.87{\times}10^{-3}}$
	CD22	−0.131	$\mathbf{7.42{\times}10^{-3}}$	−0.119	$\mathbf{2.06{\times}10^{-2}}$
	CD70	−0.105	$\mathbf{3.30{\times}10^{-2}}$	−0.102	$\mathbf{4.62{\times}10^{-2}}$
	CD79A	−0.180	$\mathbf{2.26{\times}10^{-4}}$	−0.162	$\mathbf{1.52{\times}10^{-3}}$
Monocyte	*CD86*	0.019	$7.04{\times}10^{-1}$	0.048	$3.53{\times}10^{-1}$
	CD115 (CSF1R)	−0.050	$3.12{\times}10^{-1}$	−0.026	$6.15{\times}10^{-1}$
TAM	*CCL2*	−0.225	$\mathbf{3.66{\times}10^{-6}}$	−0.204	$\mathbf{6.23{\times}10^{-5}}$
	CD68	0.063	$1.98{\times}10^{-1}$	0.085	$9.98{\times}10^{-2}$
	IL10	−0.061	$2.14{\times}10^{-1}$	−0.050	$3.27{\times}10^{-1}$
M1 Macrophage	*INOS (NOS2)*	0.092	$6.17{\times}10^{-2}$	0.087	$8.90{\times}10^{-2}$
	IRF5	−0.015	$7.66{\times}10^{-1}$	−0.022	$6.68{\times}10^{-1}$
	COX2(PTGS2)	0.098	$\mathbf{4.64{\times}10^{-2}}$	0.105	$\mathbf{4.09{\times}10^{-2}}$
M2 Macrophage	*CD163*	0.023	$6.47{\times}10^{-1}$	0.042	$4.17{\times}10^{-1}$
	VSIG4	−0.073	$1.38{\times}10^{-1}$	−0.063	$2.23{\times}10^{-1}$
	MS4A4A	−0.029	$5.52{\times}10^{-1}$	−0.015	$7.74{\times}10^{-1}$
	IRF4	−0.051	$3.02{\times}10^{-1}$	−0.028	$5.90{\times}10^{-1}$
Neutrophil	*CD66b (CEACAM8)*	0.137	$\mathbf{5.30{\times}10^{-3}}$	0.119	$\mathbf{2.06{\times}10^{-2}}$
	CD11b (ITGAM)	−0.038	$4.39{\times}10^{-1}$	−0.028	$5.87{\times}10^{-1}$
	CCR7	−0.159	$\mathbf{1.14{\times}10^{-3}}$	−0.148	$\mathbf{3.94{\times}10^{-3}}$
Natural killer cell	*KIR2DL1*	0.031	$5.26{\times}10^{-1}$	0.011	$8.31{\times}10^{-1}$
	KIR2DL3	0.146	$\mathbf{2.90{\times}10^{-3}}$	0.158	$\mathbf{2.00{\times}10^{-3}}$
	KIR2DL4	0.071	$1.48{\times}10^{-1}$	0.064	$2.14{\times}10^{-1}$
	KIR3DL1	0.049	$3.23{\times}10^{-1}$	0.058	$2.63{\times}10^{-1}$
	KIR3DL2	0.074	$1.34{\times}10^{-1}$	0.07	$1.71{\times}10^{-1}$
	KIR3DL3	0.181	$\mathbf{2.03{\times}10^{-4}}$	0.16	$\mathbf{1.84{\times}10^{-3}}$
	KIR2DS4	0.07	$1.53{\times}10^{-1}$	0.067	$1.95{\times}10^{-1}$
Dendritic cell	*HLA-DPB1*	−0.068	$1.65{\times}10^{-1}$	−0.053	$3.03{\times}10^{-1}$
	HLA-DQB1	−0.029	$5.62{\times}10^{-1}$	−0.011	$8.28{\times}10^{-1}$
	HLA-DRA	0.032	$5.20{\times}10^{-1}$	0.05	$3.29{\times}10^{-1}$
	HLA-DPA1	−0.003	$9.50{\times}10^{-1}$	0.012	$8.12{\times}10^{-1}$
	BDCA-1(CD1C)	−0.113	$\mathbf{2.15{\times}10^{-2}}$	−0.102	$\mathbf{4.63{\times}10^{-2}}$
	BDCA-4(NRP1)	−0.008	$8.70{\times}10^{-1}$	0.007	$8.99{\times}10^{-1}$
	CD11c (ITGAX)	0.055	$2.64{\times}10^{-1}$	0.08	$1.18{\times}10^{-1}$

Supplementary Table 9 **Correlation analysis between *C1GALT1* and marker genes in immune cells using TIMER** (continued)

Description	Gene markers	STAD			
		Unadjusted by purity		Adjusted by purity	
		Cor	AAAPBBB	Cor	AAAPBBB
Th1	*T-bet (TBX21)*	−0.067	1.95×10^{-1}	−0.068	1.68×10^{-1}
	STAT4	−0.009	8.49×10^{-1}	0.018	7.32×10^{-1}
	STAT1	0.194	**6.94×10^{-5}**	0.201	**8.24×10^{-5}**
	IFN-γ (IFNG)	0.095	5.20×10^{-2}	0.106	**3.90×10^{-2}**
	TNF-α (TNF)	0.003	9.56×10^{-1}	0.022	6.72×10^{-1}
Th2	*GATA3*	−0.191	**9.02×10^{-5}**	−0.179	**4.72×10^{-4}**
	STAT6	0.109	**2.70×10^{-2}**	0.099	5.36×10^{-2}
	BATF	0.047	3.45×10^{-1}	0.06	2.45×10^{-1}
	CD294 (GPR44)	0.098	**4.65×10^{-2}**	0.104	**4.36×10^{-2}**
	IL13	−0.046	3.54×10^{-1}	−0.028	5.88×10^{-1}
Tfh	*BCL6*	−0.102	**3.88×10^{-2}**	−0.091	7.72×10^{-2}
	IL21	0.083	**9.17×10^{-2}**	0.088	8.86×10^{-2}
	CD185 (CXCR5)	−0.161	**9.67×10^{-4}**	−0.155	**2.47×10^{-3}**
	CD278 (ICOS)	0.064	1.90×10^{-1}	0.096	6.12×10^{-2}
Th17	*STAT3*	0.122	**1.32×10^{-2}**	0.122	**1.71×10^{-2}**
	CD121a (IL1R1)	−0.018	7.15×10^{-1}	0.006	9.02×10^{-1}
	CD194 (CCR4)	−0.069	1.63×10^{-1}	−0.052	3.17×10^{-1}
	CD196(CCR6)	0.17	**4.87×10^{-4}**	0.18	**4.45×10^{-4}**
	IL17 (IL17A)	0.125	**1.11×10^{-2}**	0.124	**1.58×10^{-2}**
	IL21	0.083	9.17×10^{-2}	0.088	8.86×10^{-2}
	IL22	0.059	2.28×10^{-1}	0.059	2.49×10^{-1}
	IL23R	0.208	**1.92×10^{-5}**	0.212	**3.28×10^{-5}**
Treg	*FOXP3*	−0.041	4.07×10^{-1}	−0.022	6.75×10^{-1}
	CD25 (IL2RA)	0.031	5.30×10^{-1}	0.055	2.86×10^{-1}
	CCR8	0.041	4.10×10^{-1}	0.061	2.40×10^{-1}
	*STAT5*B	−0.031	5.23×10^{-1}	−0.032	5.29×10^{-1}
	TGFβ (TGFB1)	−0.25	**2.44×10^{-7}**	−0.23	**6.31×10^{-6}**
T cell exhaustion	*PD-1 (PDCD1)*	−0.157	**1.36×10^{-3}**	−0.157	**2.18×10^{-3}**
	CTLA4	−0.003	9.50×10^{-1}	0.016	7.51×10^{-1}
	LAG3	−0.09	6.66×10^{-2}	−0.09	8.01×10^{-2}
	TIM-3 (HAVCR2)	0.041	4.03×10^{-1}	0.057	2.67×10^{-1}
	*GZM*B	0.007	8.88×10^{-1}	0.021	6.83×10^{-1}

Bold fonts indicate *P*-values less than 0.05. Abbreviations: TIMER, tumor immune estimation resource; STAD, stomach adenocarcinoma; TAM, tumor-associated macrophage; Th, T helper cell; Tfh, follicular helper T cell; Treg, regulatory T cell; Cor, *r* value of Spearman correlation.
